# Leveraging a 7-Layer Long Short-Term Memory Model for Early Detection and Prevention of Diabetes in Oman: An Innovative Approach

**DOI:** 10.3390/bioengineering11040379

**Published:** 2024-04-15

**Authors:** Khoula Al Sadi, Wamadeva Balachandran

**Affiliations:** 1 Department of Electronic and Electrical Engineering Research, Brunel University London, Uxbridge UB8 3PH, UK; wamadeva.balachandran@brunel.ac.uk; 2 Information Technology Department, University of Technology and Applied Sciences-Al-Mussanha, P.O. Box 13, Muladdah 314, Oman

**Keywords:** artificial intelligence, LSTM, diabetes prediction, predictive healthcare, Oman, early detection, public health

## Abstract

This study develops a 7-layer Long Short-Term Memory (LSTM) model to enhance early diabetes detection in Oman, aligning with the theme of ‘Artificial Intelligence in Healthcare’. The model focuses on addressing the increasing prevalence of Type 2 diabetes, projected to impact 23.8% of Oman’s population by 2050. It employs LSTM neural networks to manage factors contributing to this rise, including obesity and genetic predispositions, and aims to bridge the gap in public health awareness and prevention. The model’s performance is evaluated through various metrics. It achieves an accuracy of 99.40%, specificity and sensitivity of 100% for positive cases, a recall of 99.34% for negative cases, an F1 score of 96.24%, and an AUC score of 94.51%. These metrics indicate the model’s capability in diabetes detection. The implementation of this LSTM model in Oman’s healthcare system is proposed to enhance early detection and prevention of diabetes. This approach reflects an application of AI in addressing a significant health concern, with potential implications for similar healthcare challenges relating to globally diagnostic capabilities, representing a significant leap forward in healthcare technology in Oman.

## 1. Introduction

The integration of deep learning technologies into healthcare marks a pivotal shift in the landscape of medical diagnostics, particularly in the realm of chronic metabolic disorders like diabetes. Long Short-Term Memory (LSTM) networks, a form of recurrent neural networks, have emerged as a significant innovation in this area, offering new possibilities for early detection and management of diabetes [1].

This study centres on the development and implementation of a novel 7-layer LSTM model, specifically tailored for diabetes prediction. This model represents a significant advancement in the application of deep learning for medical diagnostics, combining computational intelligence with clinical insights to create a tool of remarkable accuracy and efficiency [2].

The current research in medical diagnostics and LSTM applications reflects a growing interest in predictive healthcare analytics. Previous studies, including our own work on a prediction model for Type 2 Diabetes Mellitus in Oman using artificial neural networks and machine learning classifiers, have laid the groundwork for this research [3]. Another notable contribution in this field is our exploration of a 4D CNN model for Type 2 Diabetes screening in Oman, which shares the same dataset and pre-processing methods as the current study [4].

Despite the promising developments in this field, there are still diverging views and challenges to be addressed, particularly in the adaptation and optimization of LSTM models for specific medical applications. This study aims to contribute to this evolving field by providing a detailed analysis of the 7-layer LSTM model, focusing on its architecture, data preparation, training dynamics, and performance evaluation.

In summary, this paper not only delves into the technical aspects of LSTM models but also highlights their potential in revolutionizing diabetes prediction and screening, thereby enhancing healthcare outcomes. Our findings underscore the importance of advanced computational models in medical diagnostics and their role in ushering in a new era of enhanced disease management and patient care.

## 2. Related Studies

The integration of deep learning technologies, particularly Long Short-Term Memory (LSTM) networks, into healthcare diagnostics represents a significant advancement in the management of chronic disorders such as diabetes. This study investigates the application of LSTM networks in the early detection and management of diabetes, with a focus on a 7-layer LSTM model designed for this purpose. The model aims to process complex patient data effectively and identify crucial temporal patterns essential for accurate diabetes prediction.

The initial development in the application of LSTM models for diabetes prediction was marked by the work of Massaro et al. [5], who emphasized the importance of tailored data handling for enhanced model performance. This foundational study set the stage for subsequent research in the field. Following this, Rahman et al. [6] employed a Conv-LSTM model, achieving notable accuracy using the Pima Indians Diabetes Database (PIDD). Their study was pivotal for its sophisticated model and optimization of various parameters, setting a new standard in LSTM applications for diabetes prediction.

Bharath Kumar and Udaya Kumar [7] further advanced the field by reporting high accuracy with their Convolutional LSTM model. This model indicated the potential of LSTM in the early detection and diagnosis of diabetes. Rochman et al. [8] took a comparative approach, analysing LSTM against Gated Recurrent Unit (GRU) models. Their study highlighted the nuanced differences between these architectures, contributing to a better understanding of their respective strengths and limitations in diabetes prediction.

The evolution of LSTM in diabetes prediction also included the exploration of BiLSTM networks by Yang et al. [9], incorporating an attention mechanism and achieving significant precision and recall rates. Alex et al. [10] addressed the challenge of class imbalance in medical datasets by introducing a SMOTE-based deep LSTM model, achieving high precision and recall rates.

Arora et al. [11] showcased LSTM’s strength in analysing time-series data by forecasting diabetes progression using Continuous Glucose Monitoring data. Butt et al. [12] optimized an LSTM model for diabetes forecasting, comparing it favourably with other algorithms and reporting high accuracy. F. Iacono et al. [13] introduced personalized LSTM models for Type 1 diabetes prediction, employing the UVA/Padova simulator and focusing on modelling intra-day glucose variability and insulin sensitivity.

Srinivasu et al. [14] undertook a study to predict Type-2 Diabetes using LSTM within an RNN framework, focusing on genomic and tabular data. Despite the study’s relevance in chronic disease management, it encounters several limitations, including dataset size, architecture details, and comprehensive performance evaluation. Lastly, Jaiswal and Gupta [15] reported high accuracy with a Bi-directional LSTM model, emphasizing the robustness of LSTM models in capturing temporal relationships.

However, while recent research in diabetes prediction using LSTM models has shown promising advancements, critical gaps and areas for critique must be acknowledged and addressed. A significant concern arises from the heavy reliance on specific datasets such as the PIDD in studies by Rahman et al. [6], Jaiswal and Gupta [15], and Srinivasa et al. [14]. This reliance raises questions about the applicability of the developed models to diverse populations, potentially limiting their effectiveness.

Model complexity and real-time applications are also significant challenges. Studies like those conducted by Yang et al. [9], Alex et al. [10], and Srinivasa et al. [14] highlight the computational intensity of LSTM models. This complexity poses significant challenges for real-time deployment in clinical settings, where swift decision-making is crucial.

Generalizability issues are a notable concern in studies like Arora et al. [11] and Srinivasa et al. [14]. The effectiveness of the models developed in these studies across different diabetes types and demographic groups remains untested, raising questions about their broader applicability.

Many studies, including some referenced in this review, predominantly focus on binary classification. This approach does not fully address the complex spectrum of diabetes conditions and stages, potentially limiting the models’ clinical utility [7].

Techniques like the Synthetic Minority Over-sampling Technique (SMOTE), as employed in the study by Alex et al. [10] and Srinivasu et al. [14], address class imbalance. However, they may introduce synthetic biases that could affect the real-world applicability and fairness of the models.

The absence of multi-class classification models in some studies, including Srinivasu et al. [14], limits the ability to address more complex diabetes scenarios that involve multiple classes or stages of the disease [8].

The effectiveness of LSTM models is heavily reliant on precise data pre-processing. However, in practical healthcare scenarios, achieving accurate data pre-processing may not always be feasible or attainable [15].

While personalized models, such as those presented by Iacono et al. [13], demonstrate potential, they often rely on simulated data. This reliance may not fully capture the variability and complexity of real-world patient data, raising questions about their real-world applicability.

Addressing these critical gaps and challenges is imperative for advancing predictive models in healthcare. While LSTM models have shown promise in diabetes prediction, there are significant areas for improvement. Expanding dataset diversity, exploring multi-class classification, and addressing computational challenges are essential steps. The proposed 7-layer LSTM model with high accuracy effectively addresses these gaps. However, it must also focus on optimizing computational efficiency and validation across diverse datasets, representing different demographics and diabetes types. This model holds promise in advancing predictive modelling for diabetes, aligning with the current need for more advanced, efficient, and diverse LSTM applications in healthcare and potentially setting a new benchmark in the predictive modelling of diabetes.

## 3. Materials and Methods

**Figure 1:**
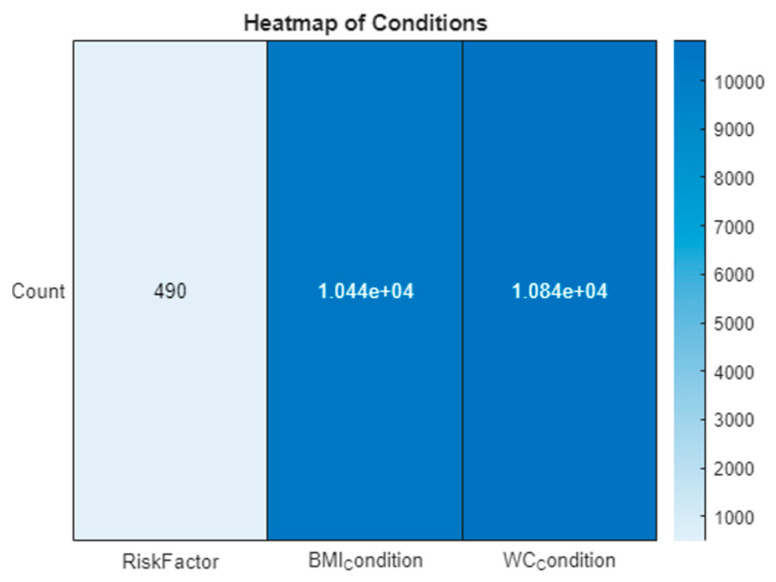
Heatmap of conditions.

This section details the methodology behind the development of a 7-layer Long Short-Term Memory (LSTM) model, focusing on its architectural design, data processing, and evaluation metrics for diabetes prediction. The model’s development, illustrated in Figure 1, highlights its structural design and operational workflow.

The Oman Screening Dataset serves as the cornerstone of our research into early diabetes prediction within the Sultanate of Oman. In this section, we provide a comprehensive overview of this dataset, shedding light on its origins, features, collection process, inclusion criteria, and its direct relevance to Oman.

### 3.1. The Oman Screening Dataset

The dataset encompasses 13,224 individual records, each detailed with 13 essential variables that were carefully chosen based on their relevance to diabetes risk assessment. These variables include demographic information (such as age and gender), anthropometric measurements (including weight, height, Body Mass Index [BMI], and Waist Circumference [WC]), clinical indicators (Total Cholesterol [T_Cholesterol], Blood Pressure [BP], Random Plasma Glucose [RPG], Fasting Plasma Glucose [FPG]), and historical data on family and personal history of diabetes. The inclusion criteria targeted individuals aged 20 years and above, specifically excluding those with prior diabetes diagnoses or who had undergone recent screenings, to focus the research on at-risk populations.

The dataset’s collection and validation processes were carried out with high precision and ethical rigor, obtaining necessary approvals and following guidelines to ensure data integrity and participant confidentiality. The collaboration with local healthcare experts and institutions not only enriched the dataset but also ensured its alignment with the health context and needs of Oman [4].

**Figure 2:**
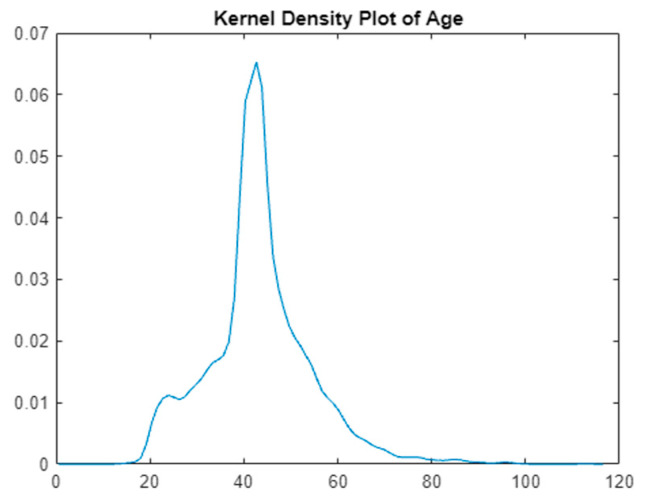
Kernel density.

To elucidate the dataset’s insights, visual analytical tools were employed. A heatmap in Figure 1 visually represents the prevalence and distribution of various risk factors across the dataset, highlighting particularly the impact of obesity-related conditions. Additionally, a kernel density plot in Figure 2 focuses on the age distribution within the dataset, pinpointing the age group most at risk for diabetes. These figures are instrumental in providing a clear visual interpretation of the data, supporting the research findings and recommendations.

The visual tools of the heatmap and kernel density plot reveal critical insights. The heatmap shows a lower occurrence of ‘RiskFactor’ conditions compared to ‘BMIcondition’ and ‘WCcondition’, emphasizing the prevalence of obesity-related conditions. The kernel density plot indicates a concentration of individuals within a specific age range, guiding focus onto the age group most at risk.

### 3.2. Model Architecture

**Figure 3:**
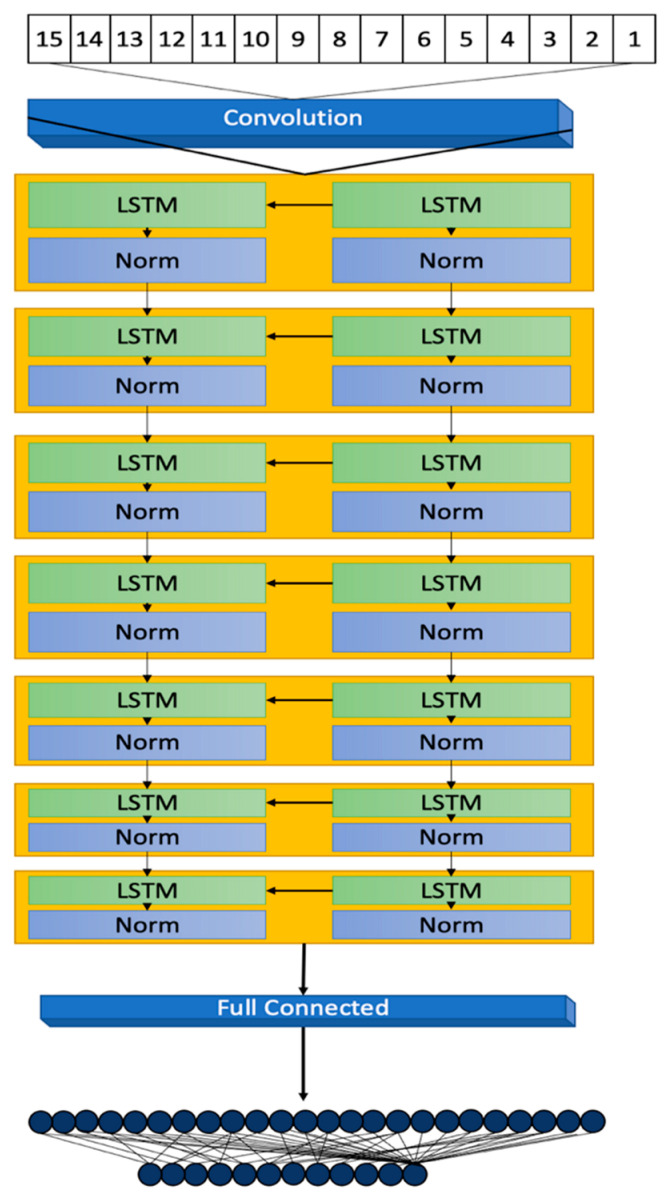
The 7-layer LSTM Architecture.

The 7-layer Long Short-Term Memory (LSTM) model, illustrated in Figure 3, is designed to meticulously process sequences of data reflective of the dynamic and complex nature typical of datasets related to diabetes. This sophisticated architecture plays a pivotal role in accurately capturing and predicting the intricate patterns and trends inherent in the data.

The architecture initiates with a sequence input layer responsible for the normalization of a diverse array of input variables. By converting disparate data points into a standardized sequence format, this layer facilitates the effective processing of data through the model’s subsequent LSTM layers. The primary inputs to the model encompass a suite of 12 features, including demographic information (Age, Gender Encoded), anthropometric measurements (Weight, Height, BMI, Waist Circumference [WC]), and clinical indicators (Total Cholesterol [T_Cholesterol], Blood Pressure [BP], Random Plasma Glucose [RPG], Fasting Plasma Glucose [FPG], Family History [FH], and Personal History [PH]). These features have been meticulously selected for their direct relevance to diabetes risk assessment.

To illustrate the model’s input and output process, consider a patient profile characterized by the following: Age = 45 years, Gender = Female, Weight = 74.5 kg, Height = 158.5 cm, BMI = 29.6, WC = 85 cm, T_Cholesterol = 4.5 mg/dL, BP = 87 mmHg, RPG = 11.6 mmol/L, FPG = 7.2 mmol/L, with a positive family history of diabetes (FH = 2) and a personal history of diabetes (PH = 1). After normalization, these inputs are processed through the model’s layers, culminating in a probabilistic score that reflects the patient’s diabetes risk.

In addition to these primary inputs, three derived conditions, identified as 13 (BMI Condition), 14 (WC Condition), and 15 (Risk Factor) in Figure 1, are calculated based on the inputs’ values to bolster the model’s predictive precision. The BMI and WC conditions are encoded according to predefined health risk thresholds, whereas the Risk Factor is a composite measure derived from the clinical indicators, offering a comprehensive view of the patient’s overall diabetes risk.

Subsequent to the sequence input layer, the model features five LSTM layers, each comprising 20 hidden units. This neural network variant is adept at learning and retaining long-term dependencies, crucial for deciphering the temporal sequences and patterns that signal diabetes risk.

The hidden units within these layers are key to identifying subtle data variations, indicative of emergent trends or health risks [16,17]. The model is deliberately designed to mitigate overfitting, ensuring robust generalization to new, unseen data while retaining the ability to learn from the training dataset.

Normalization steps interspersed between the LSTM layers are vital for training stability, standardizing the outputs from one layer before they proceed to the next. This process addresses potential scale and range discrepancies, fostering a smooth learning progression [18,19].

Concluding the architecture are a fully connected layer and a regression layer, which collectively refine the prediction of the outcome variable through mean-squared error loss computation. The thoughtful configuration of the model, including the number of hidden units, normalization type, and layer configuration, is tailored to the dataset’s specificities and the predictive task at hand. Continuous model monitoring during training is crucial to avert overfitting [20] and for hyperparameter adjustments as necessary.

Applicable to an array of machine learning endeavours beyond diabetes prediction, this model’s capability of analysing sequential and time-series data renders it suitable for varied tasks like language modelling, stock-market forecasting, and activity recognition.

The model’s depth and complexity enable the extraction of high-level abstract data features, although its training demands significant computational resources. Proper initialization and optimization techniques can mitigate the risks of vanishing or exploding gradients, underscoring the model’s powerful utility in generating accurate predictions from complex, temporal data. Its architecture, robust and adaptable, is poised to significantly contribute across a wide spectrum of applications where time dimension understanding is paramount.

### 3.3. Data Transformation and Preparation

Optimal functionality of the LSTM model is contingent upon thorough data preparation. MATLAB’s robust capabilities are harnessed to undergo a rigorous pre-processing protocol. This stage involves an assessment of missing data and the employment of a K-nearest neighbours (KNN) imputation method, which is specifically designed for diabetes-related datasets [21]. The effectiveness of this approach is corroborated by comparing it with alternative imputation techniques, ensuring the dataset is ideally conditioned for LSTM processing. These enhancements are built upon methodologies established in preceding research [4], laying a solid groundwork for our predictive modelling pursuits.

The data loading process involves importing a range of variables from a pre-processed dataset, where each variable is carefully vetted for missing values. The KNN imputation fills these gaps, drawing from the patterns inherent within the data. The processing phase then transforms the data into an array, categorizing the ‘Outcome’ variable for the LSTM’s use. Subsequent pre-processing steps include outlier removal and the encoding of risk factors, such as abnormal glucose levels and high blood pressure, into the dataset.

Statistical measures like mean, standard deviation, skewness, and kurtosis are computed to understand the data’s distribution, further informing the pre-processing strategy. Features are processed into categorical groups—such as age groups and BMI categories—then converted into numerical arrays to be incorporated into the LSTM model.

The dataset is divided into training, validation, and test sets, with the training data transformed into a sequence format suitable for the LSTM. This transformation is critical for capturing the temporal dependencies essential for accurate predictions.

### 3.4. Model Training Dynamics

The LSTM model’s training regimen is characterized by the careful calibration of several key parameters. The number of epochs, the size of the mini-batches, and the learning rate are all optimized to enhance the model’s learning trajectory. The Adam optimizer [19] is selected for its effectiveness in managing sparse gradients within large datasets. Additionally, a gradient threshold is established to prevent gradient explosion—a common obstacle in deep neural network training.

The model’s architecture, with its sequence input layer, LSTM layers, normalization layers, and a fully connected layer leading to a regression layer, is meticulously structured to capture the complex relationships within the data. Training options such as epoch count, batch size, and learning rate are meticulously set to strike a balance between adequate learning and overfitting avoidance.

By adopting these training dynamics, the LSTM model is meticulously tuned to process and learn from the dataset, with the ultimate goal of accurately predicting diabetic outcomes. The model’s training is underpinned by a rigorous approach that ensures stability and precision throughout the learning process, marking a significant step toward reliable diabetic prediction using deep learning.

### 3.5. Performance Evaluation and Metrics

The efficacy of the model is stringently assessed through an array of metrics, namely accuracy [22], precision [23], recall [24], F1 score [25], and the Receiver Operating Characteristic–Area Under Curve (ROC-AUC) value [26]. These metrics furnish a holistic view of the model’s predictive capabilities. The ROC curve, in particular, serves as a graphical representation of the model’s skill in differentiating between diabetic and non-diabetic cases. The AUC value further quantifies the model’s discriminative power between these two categories.

The inception, refinement, and validation of this LSTM model underscore the significant potential and hurdles associated with the application of advanced machine learning techniques in the domain of healthcare diagnostics. The model’s notable accuracy in diabetes prediction underscores its utility as a diagnostic tool and encourages ongoing exploration into disease prediction utilizing more sophisticated machine learning models.

In a specific example utilizing patient data, the input for the sequence input layer is organized as a sequence array from the patient’s data points: (GenderEncoded, Age, Weight, Height, BMI, Waist Circumference (WC), Total Cholesterol, Blood Pressure (BP), Random Plasma Glucose (RPG), Fasting Plasma Glucose (FPG), Family History (FH), Personal History (PH)). These values are normalized and processed through the LSTM layers for pattern recognition and risk evaluation. The model’s output, post-analysis, offers a predictive score that delineates the patient’s likelihood of being diabetic or non-diabetic. This score, alongside the aforementioned evaluation metrics, is instrumental in gauging the model’s precision and dependability.

## 4. Model Evaluation and Results

The performance evaluation of our 7-layer Long Short-Term Memory (LSTM) model in diabetes prediction involves an in-depth analysis of several key statistical metrics. Understanding what each of these metrics represents is crucial in gauging the model’s effectiveness, particularly in a clinical diagnostic setting.

### 4.1. Analysis of the Confusion Matrix and Model’s Predictive Power

The confusion matrix, an essential tool in evaluating the performance of classification models, provides valuable insights into the model’s predictive power [27]. Table 1 presents a confusion matrix comparing actual and predicted classifications for diabetic and non-diabetic cases.

(a) Specificity (100%): specificity measures the model’s accuracy in identifying non-diabetic cases. It reaches a perfect 100%, indicating that the model correctly identifies all non-diabetic cases. The absence of false positives underscores the model’s precision and accuracy. In practical terms, this means that no individual without diabetes is incorrectly diagnosed as diabetic.
(b) Precision (100%): precision assesses the model’s accuracy in predicting diabetic cases, with a rate of 100%. This exceptional precision minimizes the chances of false diabetic diagnoses. When the model predicts a positive case (diabetes), it is incredibly accurate, ensuring that individuals identified as diabetic are highly likely to have the condition.
(c) Recall (Sensitivity) (100% for Positive Class, 99.34% for Negative Class): recall evaluates the model’s ability to detect actual diabetic cases. High recall rates ensure comprehensive patient care and minimize missed diagnoses. Specifically, for the positive class (diabetic cases), the recall rate is 100%, meaning the model correctly identifies all diabetic individuals. For the negative class (non-diabetic cases), the recall rate is 99.34%, indicating that the model successfully identifies the vast majority of non-diabetic individuals.
(d) F1 Score (96.24%): the F1 Score harmonizes precision and recall, signifying a strong balance between identifying diabetic cases accurately and minimizing false positives. This balanced metric is particularly important in medical diagnostics, where both false positives and false negatives can have significant consequences. The F1 Score of 96.24% demonstrates the model’s ability to achieve both high precision and recall simultaneously.
(e) Accuracy (99.40%): the high accuracy rate reflects the model’s reliability in disease classification. An accuracy of 99.40% means that the model correctly classifies nearly all cases (both diabetic and non-diabetic), making it an effective tool for diabetes prediction.

### 4.2. Remaining Performance Metrics

**Figure 4:**
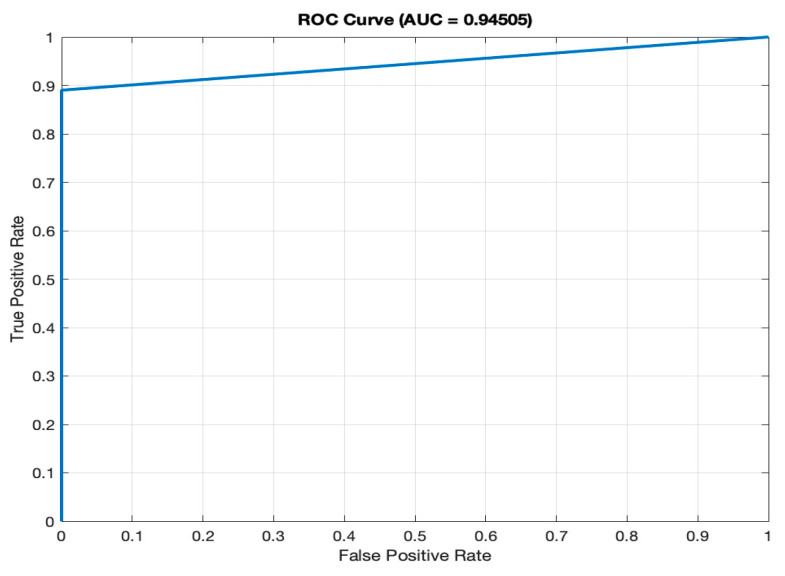
ROC Curve.

**Figure 5:**
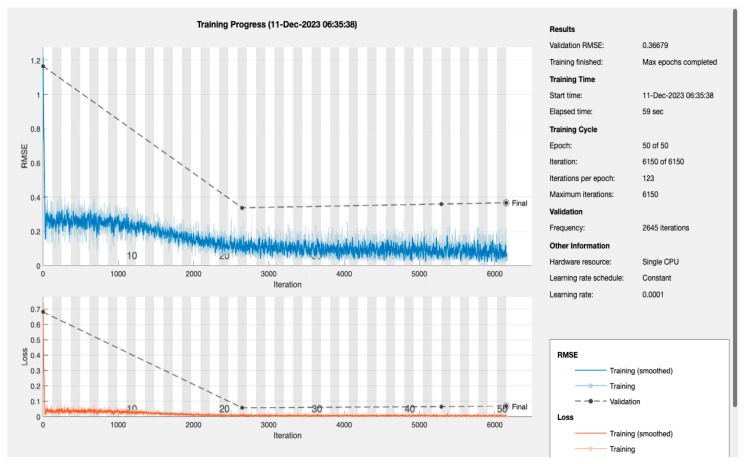
Training progress of LSTM model.

(f) AUC (94.51%): the ROC (Receiver Operating Characteristic) curve, as depicted in Figure 4, illustrates the model’s ability to differentiate between diabetic and non-diabetic classes across various thresholds. The high AUC value of 94.51% signifies superior discriminatory power, a vital characteristic for accurate classification in medical diagnostics. A high AUC value means that the model is excellent at distinguishing between individuals with diabetes and those without.

(g) The LSTM Model Training Dynamics and Efficiency: the dynamics of the LSTM model’s training process are encapsulated in Figure 5, which presents a detailed view of the Root Mean Square Error (RMSE) and loss throughout the training iterations [28]. The model’s RMSE initially shows a steep decline, reflecting a quick and significant learning phase. This rapid improvement stabilizes as the training progresses, which is expected behaviour as the model begins to converge. The final-validation RMSE value stands at an impressive 0.36679, pointing to the model’s high level of prediction accuracy. Complementary to the RMSE, the loss metrics plotted over the same iteration span show a similar trend—initially decreasing sharply before plateauing. The alignment between the training and validation loss indicates the model’s successful generalization, suggesting it can reliably extend its predictions to unseen data without significant overfitting.

The efficiency of the model’s training is also noteworthy, with the entire process being completed in just 59 s on a single CPU. This swift training time is particularly advantageous in medical settings where quick model deployment is crucial. When considered alongside the AUC of 94.51%, previously discussed, it becomes clear that the LSTM model is not only quick to train but also offers a high degree of precision, essential for medical diagnostic applications where accurate and timely predictions can be critical.

(h) Alpha Values Assessment:
  In further examining the LSTM model’s performance, particular attention was given to the “Alpha” values [29], which signify the model’s internal class-weighting mechanism. These values critically influence the balance between sensitivity (true positive rate) and specificity (true negative rate), affecting overall predictive performance.
  - For non-diabetic (0): Alpha = 1. This allocation signals the model’s emphasis on accurately identifying non-diabetic instances, aiming to eliminate false positives. Such prioritization is vital to prevent misdiagnosis, avoiding unnecessary intervention for those incorrectly identified as diabetic.
  - For diabetic (1): Alpha = 0. This setting suggests a strategic decision to adjust the model’s focus. It does not imply a neglect of diabetic case detection, but rather reflects a calibrated approach to optimize overall performance. This may address specific challenges like dataset imbalance or the objective of achieving a balanced sensitivity and specificity, ensuring that the model remains highly effective while minimizing potential misclassifications.

The implementation of distinct Alpha values for each class underscores the model’s sophisticated approach to managing diagnostic complexities. By differentiating the importance attached to each outcome, the model adeptly navigates the delicate balance of reducing false positives and capturing true diabetic cases without omission. This tailored adjustment is crucial in medical diagnostics, where the stakes attached to every prediction are high, influencing subsequent care and treatment pathways.

Moreover, the strategic use of Alpha values highlights the LSTM model’s advanced capability of adapting to the nuanced demands of medical diagnostics. It showcases the potential of deep learning techniques to significantly advance medical science, particularly in achieving high accuracy in disease prediction and classification.

### 4.3. Interpretation and Clinical Relevance

The analysis of the confusion matrix results, combined with the performance metrics, highlights the proficiency and clinical relevance of our LSTM model in diabetes prediction. The absence of false positives, a high specificity, and recall rates underscore the model’s precision and sensitivity, essential attributes in healthcare diagnostics.

These results demonstrate the model’s accuracy and reliability, making it a valuable tool in the medical field. The model’s performance is exceptional in terms of correctly identifying diabetic and non-diabetic cases, achieving both high precision and recall rates. Its AUC score further confirms its ability to make accurate distinctions, and its overall accuracy demonstrates its trustworthiness.

The LSTM model’s exceptional performance, as evidenced by its high accuracy, precision, recall, F1 Score, AUC, and negligible Alpha error, positions it as a sophisticated diagnostic tool. This thorough evaluation lays a solid foundation for its application in broader medical scenarios, promising to improve patient care and treatment outcomes.

## 5. Discussion

The comparative analysis of various LSTM models for diabetes prediction, as detailed in Table 2, provides a comprehensive evaluation of the performance metrics across different studies, highlighting the advancements and challenges in this area of medical diagnostics. This discussion aims to contextualize the results of the 7-layer LSTM model developed in our study within the broader landscape of LSTM applications in diabetes prediction.

The Conv-LSTM model [4], which utilized the Pima Indians Diabetes Database [30], achieved an impressive accuracy of 97.26%, although specific metrics like precision, recall, and sensitivity were not disclosed. This indicates a strong baseline performance for LSTM models in diabetes prediction. The LSTM-AR model [15], with its implementation on an ERP platform, demonstrated notable precision and recall rates but showed a disparity in recall rates for positive and negative classes, indicating potential areas for improvement in model balance.

A comparison between LSTM and GRU models [8,13] revealed that GRU might offer better accuracy in certain contexts, suggesting that the choice of model architecture could be crucial depending on the specific nature of the diabetes data being analysed. The BiLSTM with the Attention model [9], which employed EHRs for prediction, reportedly achieved higher precision and recall than traditional methods, although exact figures were not specified, highlighting the potential of attention mechanisms in enhancing LSTM model performance.

The application of SMOTE in the Deep LSTM model [10] to address class imbalance and its resulting high accuracy of 99.64% underscores the importance of addressing data pre-processing challenges in model development. Similarly, the LSTM model for Continuous Glucose Monitoring (CGM) [11], with an average RMSE of 4.02, points to the growing trend of LSTM applications in continuous data monitoring scenarios.

The BLSTM model [15] emphasized sensitivity, achieving high rates of recall for the positive class and specificity, indicating its effectiveness in correctly identifying diabetic cases. This is particularly relevant in medical diagnostics, where the cost of false negatives can be high.

In contrast to these models, the 7-layer LSTM model developed in this study demonstrated unparalleled performance with a precision and recall rate of 100%, a specificity of 100%, and an accuracy of 99.40%. This exemplary performance, especially in terms of sensitivity and specificity, positions our model as a highly effective tool in diabetes prediction, surpassing the benchmarks set by other LSTM models.

This analysis not only highlights the strengths of the 7-layer LSTM model but also sheds light on the varied applications and potential of LSTM models in diabetes prediction. The high accuracy and reliability of our model suggest significant potential for improving diabetes diagnosis, leading to more accurate and early detection of the disease, which is crucial for patient outcomes. The results also indicate the importance of model architecture, data pre-processing, and the need for balancing precision and recall in model development.

The comparative performance of the 7-layer LSTM model opens up new possibilities for enhancing diagnostic accuracy and patient care in the medical field. Future research should focus on broadening the application scope of this model, exploring its integration in clinical practice, and assessing its adaptability to diverse datasets and real-world medical settings.

## 6. Conclusions

This study’s analysis of the 7-layer Long Short-Term Memory (LSTM) model demonstrates a notable advancement in diabetes prediction using predictive healthcare analytics. The model’s complex architecture, combined with rigorous data preparation and strategic training using MATLAB and the Adam optimization algorithm, contributes to its high efficacy in diagnosing diabetes.

Its performance evaluated using accuracy, precision, recall, F1 score, and ROC-AUC, shows exceptional capability in distinguishing between diabetic and non-diabetic cases, with remarkable precision and recall rates. This positions the model as a highly effective diagnostic tool. The multi-layered structure of the LSTM model enhances its accuracy in predicting diabetes.

Compared to other LSTM-based models, the 7-layer LSTM model shows superior performance, indicating its potential to significantly improve diabetes diagnosis. The study suggests future applications of the model on diverse datasets and in real-world clinical settings to validate its effectiveness and broaden its use in diabetes care.

In summary, the 7-layer LSTM model stands as a significant contribution to medical diagnostics, offering a powerful tool for early diabetes detection and prevention. Its integration into clinical practice could revolutionize personalized healthcare and patient management, marking a new era in applying machine learning in healthcare diagnostics.

## Figures and Tables

**Table 1:** Confusion Matrix.

Actual vs. Predicted	Non-Diabetic (0)	Diabetic (1)
Non-Diabetic (0)	2424	0
Diabetic (1)	16	205

**Table 2:** Comparative Performance of Various LSTM Models in Diabetes Prediction.

Model Description	Precision	Recall (Positive Class)	Recall (Negative Class)	Accuracy	AUC	Sensitivity	Specificity	F1 Score	Additional Notes
Conv-LSTM [7]	Not specified	Not specified	Not specified	97.26%	N/A	Not specified	Not specified	Not specified	Used Pima Indians Diabetes Database
LSTM-AR [15]	75.73%	83.66%.	49.38%.	71.79%	N/A	83.66%	49.38%	Not specified	Implemented on ERP platform
LSTM vs GRU [8]	Not specified	Not specified	Not specified	GRU better	N/A	Not specified	Not specified	Not specified	RMSE used for comparison
LSTM and GRU [13]	Not specified	Not specified	Not specified	Not specified	Sensitivity, Specificity, F1-score, MCC	Not specified	Not specified	Not specified	Genomic data used for prediction
BiLSTM with Attention [9]	Higher than traditional	Not specified	Not specified	Not specified	Precision and Recall	Not specified	Not specified	Not specified	Utilized EHRs for prediction
SMOTE-based Deep LSTM [10]	Not specified	Not specified	Not specified	99.64%	N/A	Not specified	Not specified	Not specified	Employed SMOTE for class imbalance
LSTM for CGM [11]	Not specified	Not specified	Not specified	Not specified	Average RMSE: 4.02	Not specified	Not specified	Not specified	Predicted blood glucose trends
BLSTM [15]	Not specified	96%	91%	94%		Not specified	91%	93%	Sensitivity emphasized in the study
7-layer LSTM (this study)	100%	100%	99.34%	99.40%	94.51%	100%	100%	96.24%	High accuracy and reliability

The following is a key to understanding the abbreviations used in the table. N/A: Not Applicable, GRU: GRU performed better than LSTM in terms of accuracy, RMSE: Root-Mean-Square Error, EHRs: Electronic Health Records, CGM: Continuous Glucose Monitoring, MCC: Mathew’s Correlation Coefficient.

## Data Availability

The uniquely constructed Oman Diabetes Type II Screening Dataset, which substantiates the findings of this study, can be made available upon reasonable request by contacting the corresponding author.

## References

[1] Shickel B., Tighe P.J., Bihorac A., Rashidi P. (2018). Deep EHR: A Survey of Recent Advances in Deep Learning Techniques for Electronic Health Record (EHR) Analysis. IEEE J. Biomed. Health Inform..

[2] Choi E., Schuetz A., Stewart W.F., Sun J. Doctor AI: Predicting Clinical Events via Recurrent Neural Networks. Proceedings of the Machine Learning for Healthcare Conference.

[3] Al Sadi K., Balachandran W. (2023). Prediction Model of Type 2 Diabetes Mellitus for Oman Prediabetes Patients Using Artificial Neural Network and Six Machine Learning Classifiers. Appl. Sci..

[4] Al Sadi K., Balachandran W. (2023). Revolutionizing Early Disease Detection: A High-Accuracy 4D CNN Model for Type 2 Diabetes Screening in Oman. Bioengineering.

[5] Massaro A., Maritati V., Giannone D., Convertini D., Galiano A. (2019). LSTM DSS Automatism and Dataset Optimization for Diabetes Prediction. Appl. Sci..

[6] Rahman M., Islam D., Mukti R.J., Saha I. (2020). A deep learning approach based on convolutional LSTM for detecting diabetes. Comput. Biol. Chem..

[7] Chowdary P.B.K., Udaya R. (2021). An Effective Approach for Detecting Diabetes using Deep Learning Techniques based on Convolutional LSTM Networks. Int. J. Adv. Comput. Sci. Appl..

[8] Rochman E.M.S., Suprajitno H., Rachmad A., Nindyasari R., Rachman F.H. Comparison of LSTM and GRU in Predicting the Number of Diabetic Patients. Proceedings of the 2022 IEEE 8th Information Technology International Seminar (ITIS).

[9] Yang Y., Zheng X., Ji C. Disease prediction model based on bilstm and attention mechanism. Proceedings of the 2019 IEEE International Conference on Bioinformatics and Biomedicine (BIBM).

[10] Alex S.A., Jhanjhi N.Z., Humayun M., Ibrahim A.O., Abulfaraj A.W. (2022). Deep LSTM Model for Diabetes Prediction with Class Balancing by SMOTE. Electronics.

[11] Arora S., Kumar S., Kumar P. Implementation of LSTM for Prediction of Diabetes using CGM. Proceedings of the 2021 10th International Conference on System Modelling & Advancement in Research Trends (SMART).

[12] Butt U.M., Letchmunan S., Ali M., Hassan F.H., Baqir A., Sherazi H.H.R. (2021). Machine Learning Based Diabetes Classification and Prediction for Healthcare Applications. J. Healthc. Eng..

[13] Iacono F., Magni L., Toffanin C. Personalized LSTM models for glucose prediction in Type 1 diabetes subjects. Proceedings of the 2022 30th Mediterranean Conference on Control and Automation (MED).

[14] Srinivasu P.N., Shafi J., Krishna T.B., Sujatha C.N., Praveen S.P., Ijaz M.F. (2022). Using Recurrent Neural Networks for Predicting Type-2 Diabetes from Genomic and Tabular Data. Diagnostics.

[15] Jaiswal S., Gupta P. (2023). Diabetes Prediction Using Bi-directional Long Short-Term Memory. SN Comput. Sci..

[16] Hochreiter S., Schmidhuber J. (1997). Long short-term memory. Neural Comput..

[17] Pascanu R., Mikolov T., Bengio Y. On the difficulty of training recurrent neural networks. Proceedings of the International Conference on Machine Learning.

[18] Ba J.L., Kiros J.R., Hinton G.E. (2016). Layer normalization. arXiv.

[19] Kingma P., Ba J. (2014). Adam: A Method for Stochastic Optimization. arXiv.

[20] Ashiquzzaman A., Tushar A.K., Islam M.R., Shon D., Im K., Park J.-H., Lim D.-S., Kim J. (2018). Reduction of overfitting in diabetes prediction using deep learning neural network. IT Convergence and Security 2017.

[21] MathWorks, Impute Missing Data Using Nearest-Neighbor Method—MATLAB Knnimpute. MathWorks United Kingdom. https://uk.mathworks.com/help/bioinfo/ref/knnimpute.html.

[22] Lin C.-C., Lai M.-S., Syu C.-Y., Chang S.-C., Tseng F.-Y. (2005). Accuracy of diabetes diagnosis in health insurance claims data in Taiwan. J. Formos. Med. Assoc..

[23] Hattersley A.T., Patel K.A. (2017). Precision diabetes: Learning from monogenic diabetes. Diabetologia.

[24] Willaing I., Rogvi S.Á., Bøgelund M., Almdal T., Schiøtz M. (2013). Recall of HbA1c and self-management behaviours, patient activation, perception of care and diabetes distress in Type 2 diabetes. Diabet. Med..

[25] Ayon S.I., Islam M.M. (2019). Diabetes prediction: A deep learning approach. Int. J. Inf. Eng. Electron. Bus..

[26] Lugner M., Rawshani A., Helleryd E., Eliasson B. (2024). Identifying top ten predictors of type 2 diabetes through machine learning analysis of UK Biobank data. Sci. Rep..

[27] Mijwil M.M., Aljanabi M. (2023). A Comparative Analysis of Machine Learning Algorithms for Classification of Diabetes Utilizing Confusion Matrix Analysis. Baghdad Sci. J..

[28] Nabi M., Wahid A., Kumar P. (2017). Performance Analysis of Classification Algorithms in Predicting Diabetes. Int. J. Adv. Res. Comput. Sci..

[29] Radja M., Emanuel A.W.R. (2019). Performance evaluation of supervised machine learning algorithms using different data set sizes for diabetes prediction. Proceedings of the 2019 5th International Conference on Science in Information Technology (ICSITech).

[30] El Idrissi T., Idri A., Karaca Y. (2020). Computational Science and Its Applications—ICCSA 2020. International Conference on Computational Science and Its Applications, Proceedings of the ICCSA 2020, Cagliari, Italy, 1–4 July 2020.

